# How do SYMPtoms and management tasks in chronic heart failure imPACT a person's life (SYMPACT)? Protocol for a mixed‐methods study

**DOI:** 10.1002/ehf2.13010

**Published:** 2020-09-17

**Authors:** Rosalynn C. Austin, Lisette Schoonhoven, Alison Richardson, Paul R. Kalra, Carl R. May

**Affiliations:** ^1^ School of Health Sciences, Faculty of Environmental and Life Sciences University of Southampton Southampton UK; ^2^ Department of Cardiology Portsmouth Hospitals University NHS Trust Hampshire UK; ^3^ National Institute for Health Research (NIHR) Applied Research Collaboration (ARC) Wessex Southampton UK; ^4^ Julius Center for Health Sciences and Primary Care University Medical Center Utrecht, Utrecht University Utrecht The Netherlands; ^5^ Clinical Academic Facility University Hospital Southampton NHS Foundation Trust Tremona Road Southampton UK; ^6^ Institute of Health and Wellbeing, College of Medical, Veterinary and Life Sciences University of Glasgow and the University of Portsmouth Glasgow UK; ^7^ Faculty of Public Health and Policy London School of Hygiene and Tropical Medicine London UK

**Keywords:** Chronic heart failure, Burden of treatment, Symptom burden, Self‐care, Mixed methods, Study design

## Abstract

**Aims:**

Patients with chronic heart failure (CHF) struggle to follow self‐care plans, which may lead to worsening illness and poor quality of life. Burden of treatment (BoT) describes this workload and its impact on patients' lives. Suggesting the balance between a patient's treatment workload and their capability to manage it is crucial. If BoT is reduced, self‐care engagement and quality of life may improve. This article describes the SYMPACT study design and methods used to explore how symptoms and management tasks impact CHF patients' lives.

**Methods and results:**

We used a sequential exploratory mixed‐methods design to investigate the interaction between symptoms and BoT in CHF patients.

**Conclusions:**

If symptoms and BoT are intrinsically linked, then the high level of symptoms experienced by CHF patients may lead to increased treatment burden, which likely decreases patients' engagement with self‐care plans. SYMPACT may identify modifiable factors to improve CHF patients' experience.

## Introduction

Patients with chronic heart failure (CHF) often struggle to adhere to self‐care expectations.^1^ Non‐compliance with self‐care is suggested as a contributor to poor outcomes in CHF and is attributed to self‐care that is neither sufficient nor effective.^2^ Burden of treatment (BoT) is composed of dynamic states of workload (time and energy required to treat and manage a condition) and individual capacity (factors that alter ability to do work).^3^, ^4^, ^5^, ^6^, ^7^ Overwhelming treatment burden may be associated with adverse clinical outcomes.^4^, ^6^ Patient responsibility and engagement with self‐care adding to BoT are not unique to CHF.^7^ Clinical pathways and personal capacity appear to influence BoT in lung cancer and COPD.^8^ BoT appears to be exacerbated by the level of support provided by health care systems and socio‐economic disadvantages in end‐stage kidney disease.^9^ How symptoms interact with burden of treatment has yet to be investigated.

CHF is a life‐limiting syndrome, and patients experience persistent, progressive, and debilitating symptoms such as breathlessness, fatigue, and oedema, compromising their quality of life (QoL) despite optimized clinical treatment plans.^10^ Complete elimination of symptoms is unlikely, yet there are likely treatment options that could help reduce symptoms. Lower symptom burden is associated with improved functional ability and better self‐care engagement.^11^ While the theory of BoT acknowledges symptom burden, it argues that symptoms are theoretically distinct. Examination of the literature, clinical observations, and patient and public involvement (PPI) suggests that the symptoms in CHF may directly interact with BoT.^12^ In this study, possible interactions between BoT and symptom burden will be measured quantitatively and explored qualitatively through interviews, facilitating a deeper understanding of how patients with CHF experience burden of treatment. This paper outlines the design of the study: ‘How do SYMptoms and management tasks in chronic heart failure imPACT a patient's life (SYMPACT)?’ SYMPACT will examine and explore the interaction of symptoms experienced by patients with CHF with BoT. The authors will test and explore the hypothesis (aims) that symptoms are intrinsically linked with BoT; that is, patients with lower reported symptoms will report lower BoT, and patients with higher reported symptoms will report higher BoT. This will be confirmed in how patients describe their experience. The research questions are as follows: (Phase I) Is there a relationship between quantitatively measured symptoms and BoT? (Phase II) What is the perspective of patients with CHF on their experienced symptom burden and BoT? (Phase III) How do symptoms interact with BoT in CHF?

## Study design

**FIGURE 1:**
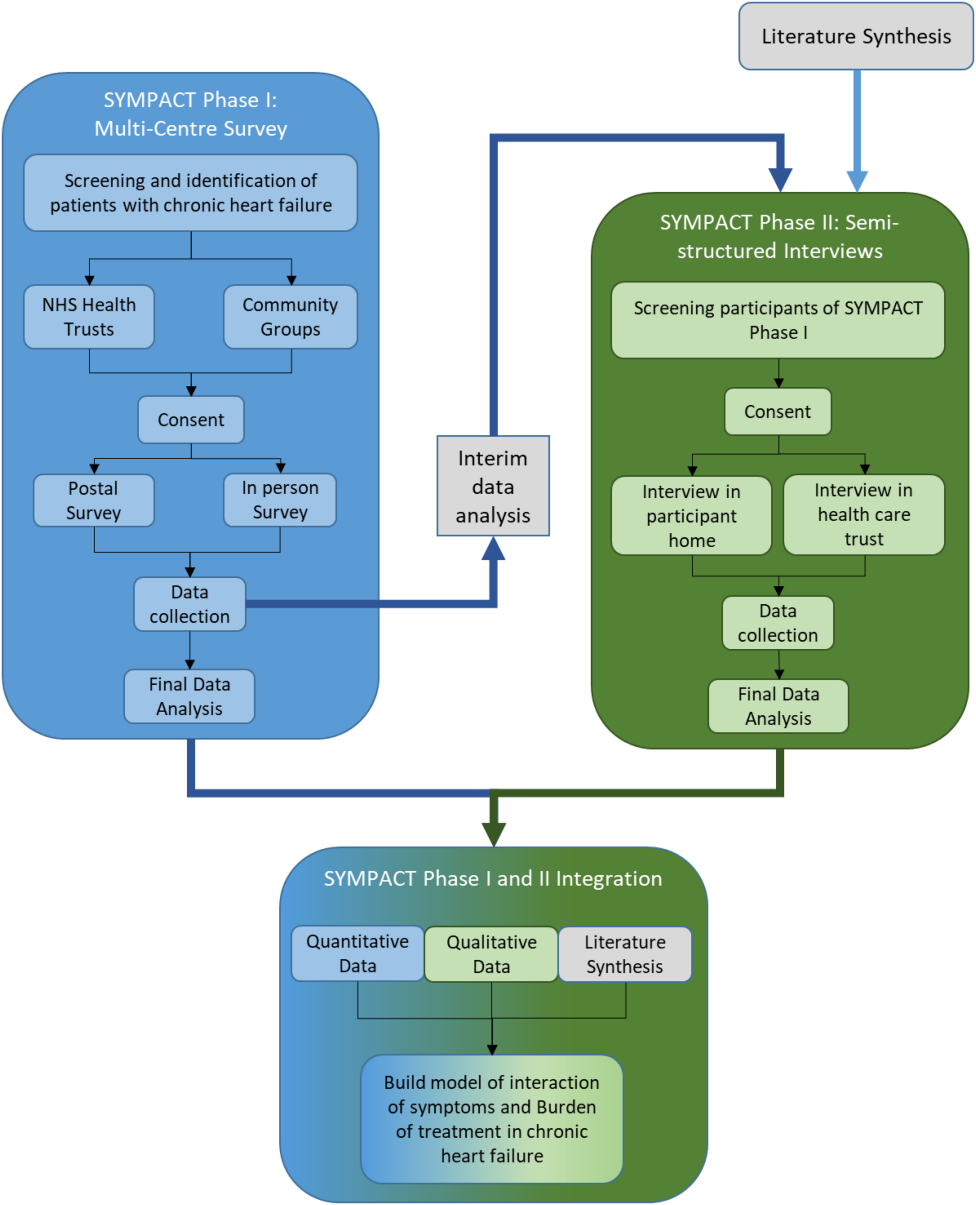
SYMPACT study design overview: flowchart illustrating the phases of SYMPACT and how they inform each other.

SYMPACT is a sequential explanatory mixed‐methods study (*Figure* *1*); this methodology promotes the exploration of results in a quantitative study.^13^, ^14^ Qualitative results expand on insights derived from quantitative results,^14^ facilitating deeper explanations of observed statistical relationships. Combining the SYMPACT study results with a qualitative literature synthesis^15^ enables the adductive analysis approach^16^ to form a conceptual model of the interaction between BoT and symptom burden in CHF.

SYMPACT adheres to the Declaration of Helsinki and has been reviewed and approved by the University of Southampton Ethics Committee (ERGO: 41287) and the Nottingham HRA1 Research Ethic Committee, Health Research Authority (MREC: 18/EM/0339, IRAS: 247773).

### Phase I: multi‐centre survey of symptoms and burden of treatment in chronic heart failure

Phase I of SYMPACT will test the following hypothesis: symptom burden reported by CHF patients will be correlated with reported domains of BoT.

#### Design

A single time point survey using three validated questionnaires is used.

#### Participants

This is a multi‐centre study across primary and secondary care National Health Service (NHS) health trusts in the UK. English‐speaking adults with CHF (minimum of 6 months), and in the investigators' opinion are not cognitively impaired, will be invited to complete the questionnaires. Patients with heart transplant or who are receiving palliative care will be excluded.

#### Methods

Following informed consent, health information will be collected: (i) demographics (age, gender, ethnicity, marital status, and living situation), (ii) CHF characteristics [diagnosis date, aetiology, New York Heart Association (NYHA) class, ejection fraction, and clinical evaluations], and (iii) personal health information (health issues, medications, and hospitalizations).

Patients will be invited to complete three validated questionnaires: Heart Failure Symptom Survey (HFSS),^17^ Minnesota Living with Heart Failure Questionnaire (MLHFQ),^18^ and the Patient Experience with Treatment and Self‐Care (PETS).^19^

- HFSS is a reliable disease‐specific evaluation of heart failure signs and symptoms.^17^ It measures the frequency, severity, and impact of 14 symptoms, where the higher score equates to more severe experience.
- MLHFQ captures key physical, emotional, social, and mental dimensions of QoL in a brief questionnaire.^20^ Lower scores on the MLHFQ relate to better health related QoL.
- PETS describes the work of illness, facilitating tools, and exacerbating factors involved in self‐care of generic chronic illness. It is a 48‐item questionnaire quantifying the patient experience of BoT across nine domains.^19^

#### Sample size

Sample size calculation is based on estimating the correlation between the above measures, to within a pre‐specified precision (as defined by the 95% confidence interval). A width of 0.2 was chosen as a balance between the practical considerations and the ability to draw useful conclusions on the observed correlations. Pearson's correlation power calculation formula^21^ suggests that a sample of size 350 will achieve this for any value of correlation.

#### Data analysis

Descriptive statistics will be used to summarize the study participants' personal, CHF, and health characteristics. Patients' questionnaire responses will be described across gender, age, number of health issues and medications, and NYHA class and according to left ventricular ejection fraction.

A scatter plot will be used to visually assess the relationship between reported symptom burden (measured by HFSS and MLHFQ) and BoT (measured by PETS); this will guide the choice of a suitable correlation tool (e.g. Pearson's or Spearman's) to summarize the relationship between the variables. Correlations will be presented with 95% confidence intervals.

An interim analysis will occur at about the halfway point. These results will be used to adapt the interview schedule, with specific probes to be used in Phase II.

#### Limitations

Limiting the sample to only three hospitals in a single county in the UK and patients who only speak English may reduce the generalizability of the results. Further, limiting the sample to CHF patients without heart transplants will ensure a focus to the generic lived experience of CHF. These limitations were thought to be reasonable owing to limited availability of translated versions of the validated questionnaires and the aim of the project.

### Phase II: semi‐structured interviews

CHF patients who complete Phase I will be invited to take part in a semi‐structured interview. The aim of this phase is to explore their experiences of living with CHF and elaborate on their questionnaire responses.

#### Design

Semi‐structured interviews will be conducted at either a health care facility or the participant's home, according to patient choice. Using the interim results of the questionnaires as probes in the interview should encourage in‐depth description of experience of BoT, thereby substantiating the results from Phase I or providing explanation for differences observed.

#### Participants

Phase I patients are eligible to participate in Phase II. Phase II will begin after the completion of the interim analysis.

#### Sampling

Sample size will be defined by data saturation when three consecutive interviews do not generate any new information.^22^ A maximum variation sampling technique will be used, ensuring that the Phase II sample is representative of the Phase I sample population.

#### Methods

Interviews will be audio recorded, and the interviewer may take field notes.

SYMPACT Phase II interview schedule is based on previous interview questions developed by other researchers interested in BoT^5^, ^23^, ^24^, ^25^, ^26^ and cover domains measured by PETS (Data *S1*: Interview schedule for Phase II). Further probing questions developed from Phase I interim results will be added to the interview schedule. Following the interview, researcher reflections on the interview will be audio recorded to promote transparency and reflexivity.

#### Data analysis

All interviews and researcher reflections will be transcribed. Field notes, where appropriate, are converted into Word documents. All participant identifiers in the interview data will be coded using their participant unique study number, assigned in Phase I.

Analysis of the interviews will follow an adapted form of Thomas and Harden's methods for thematic synthesis.^27^ With the use of NVIVO (QRS Internationals), the interview transcripts and researcher notes will be examined line by line for symptom terminology. These will form the initial nodes. Coding credibility will be achieved through member checking (PPI, clinical expert, and co‐authors).^28^ Each symptom node will be read in its context and a descriptive theme created. Finally, descriptive themes will be examined for similarities to the ‘a priori’ BoT framework. Constant comparison, reflexivity, and discussion with co‐researchers will increase rigour and trustworthiness.^29^

The a priori BoT framework incorporates the following: (1) *workload*: involving the effort required to enact tasks (technical and logistic), alter relationships (activate support and seek assistance), and evaluate outcomes of treatments (understanding and evaluation).^5^, ^23^ (2) *Individual capacity*: Encompassing an individual's abilities, their resources, and their readiness to address the workload. Including consideration of a patient's physical function, cognitive function, emotional status, socio‐economic resources, social support networks, literacy, culture, and beliefs.^6^ (3) *Impact*: Alterations to the patient's perception of self and their role. Including factors that make adhering to treatment plans more difficult.^5^, ^7^

### Phase III: data integration

Data integration for SYMPACT was planned in the study design. The validated questionnaires used were chosen according to theoretical similarities to BoT theory (detailed in *Table* *1*). The data from Phase I and Phase II will be integrated to inform the understanding of how symptoms interact with BoT in CHF. Symptom nodes and descriptive themes (Phase II) will be transformed through content analysis^30^ and compared with the symptom burden scores (HFSS and MLHFQ) and BoT scores (PETS) from Phase I, providing greater depth and insight to the patient experience. Through constantly comparing codes to the data,^13^ the descriptive themes will be refined and explored for points of interaction with the a priori BoT framework. This process should provide qualitative narratives to build on the statistical results from Phase I.

**TABLE 1:** Data integration overview for SYMPACT

A priori BoT framework	Phase I (Quantitative) variable measured	Phase II (Qualitative) question theme
*Workload*		
Enact tasks	Clinical demand^a^ PETS domains^b^	What tasks are performed in managing chronic heart failure? Does anyone help with this?
Alter relationships		
Evaluate outcomes		
*Individual capacity*		
Individual's ability	Clinical characteristics^c^ Personal characteristics^d^ HFSS total score	What makes managing chronic heart failure more difficult or easy?
Resources and readiness		
*Impact*		
Alterations to self and role	MLHFQ total score HFSS interference scores^e^ PETS domains^b^	How does the work of managing chronic heart failure impact their life?
Factors influencing adherence		

^a^ Clinical demands: number of health issues, number of medications, hospitalizations within a year.

^b^ PETS domains: sub‐scores in PETS by work (e.g. medication management and attending appointment), facilitating tools, and exacerbating factors involved in self‐care.

^c^ Clinical characteristics: NYHA classification, aetiology, ejection fraction, CHF type, and years since diagnosis.

^d^ Personal characteristics: age, gender, marital status, and living situation.

^e^ HFSS interference scores: sub‐scale in HFSS that captures reported interference with physical activity and enjoyment of life.

#### Patient and public involvement

The PPI group (members of the Patient Research Ambassadors' from the Portsmouth Hospitals University NHS Trustl) comprised members of the public with a variety of chronic conditions, including carers of people with CHF. They stated that SYMPACT asks a valuable question that they identified as being important to their experience as patients and carers. They approved of the study design, as it would encourage participants to provide more in‐depth information not covered by the questionnaires in this emerging field, which was of great importance to them, as they felt that often patient questionnaires do not ask the questions that are important to them. They provided guidance around study design elements that helped to decrease the study burden for participants. They assisted in the production of the participant and public‐facing documents as well as refining and rephrasing interview questions for Phase II. They agreed to help promote SYMPACT, perform credibility checks (part of data analysis plan for Phase II), and assist in result dissemination. This group is and continues to be an integral part to this project, and the primary author holds regular meetings with them keeping them apprised of progress and to encourage their input.

## Discussion

Patients with CHF have high symptom burden and are expected to self‐manage an illness with poor QoL and high rates of morbidity and mortality. Despite marked progress with medical and device treatments, readmission rates due to perceived non‐adherence remain high and QoL are poor. CHF prevalence is expected to rise, and the current demands on health care systems are already high. BoT provides a different perspective for examining the patient experience. It is thought that overwhelming treatment burden can lead to poor engagement with self‐care, which may contribute to poor outcomes. SYMPACT proposes a detailed measurement and exploration of treatment burden in patients with CHF. By exploring if symptoms interact with BoT, modifiable factors may be highlighted, providing the starting point for a patient‐focused intervention. Further, as there are similarities in self‐care expectations between CHF and other chronic illnesses, this study may also offer transferable knowledge to the understanding of BoT across multiple chronic illnesses.

## Conflict of Interest

Professor Alison Richardson is a National Institute for Health Research (NIHR) Senior Investigator.

## Funding

This work was completed as a part of a fully funded Clinical Academic Doctoral Fellowship at the University of Southampton, Portsmouth Hospitals University NHS Trust, and the National Institute for Health Research (NIHR) Applied Research Collaboration (ARC) Wessex. This article is independent research funded in part by the NIHR ARC Wessex. The views expressed in this publication are those of the author(s) and not necessarily those of the National Institute for Health Research, NHS or the Department of Health and Social Care.

## Ethics Statement

Ethics approval was received from the UK Health Research Authority (MREC: 18/EM/0339) and University of Southampton (ERGO: 41287). ISRCTN11011943.

## Supporting information


**Data S1.** Interview schedule for Phase II
