# Supplementary material for: How do SYMPtoms and management tasks in chronic heart failure imPACT a person's life (SYMPACT)? Protocol for a mixed‐methods study
Source: ESC Heart Fail. 2020 Sep 17;7(6):4472–7. doi: 10.1002/ehf2.13010 (PMC7754908; doi:10.1002/ehf2.13010)
Supplement: Supplementary file 1 — Data S1. Interview schedule for Phase II [file EHF2-7-4472-s001.doc]

| **BoT**  **Domain** | **Theme** | **SYMPACT Phase II Interview schedule** |
| --- | --- | --- |
| **Introduction** | Introduce the research ideas and set the context for the interview | - Introduce self and study - Purpose and length of the interview - Procedures of the interview - Invite and receive informed verbal and written consent (voluntary participation, right to stop to have a break and to withdraw, confidentiality, recording the interview, any questions) |
| **Workload** | Illness management and monitoring | - Would you tell me what it is like to live with your heart condition? *Probe around symptoms using HFSS responses.* - Can you tell me what you do at home to take care of your heart condition? *Prompts: Take medication, monitor condition, attend appointments, lifestyle behaviours, etc…* - How do you monitor your heart condition? *Probe around self-care tasks (use MLHFQ responses)* - Are there any specific symptoms that you keep track of? How? Why? *Prompt: explore the symptoms further and if they believe related to heart failure or other co-morbidity.* |
|  | Assigned work from healthcare professional | - Can you tell me about how you go about doing the tasks that your doctor/nurse wants you to do for your heart condition? Does anyone help you? What makes this harder? *Probe on symptoms, emotions, knowledge, use examples they reported in their questionnaires* - Are there things you do that make doing all these tasks easier? *Prompts: pill box, calendar.* |
|  | Health literacy | - How do you find out more information about your heart condition? *Prompts: Health care provider, GP, magazine, support group, others with your condition.* What makes you motivated to find out more about your condition? What makes it harder? |

| **BoT**  **Domain** | **Theme** | **SYMPACT Phase II Interview schedule** |
| --- | --- | --- |
| **Capacity** | Individual ability factors; helping or hindering engagement in the work of illness | - If you picture a set of scales, on the one side, you have your heart condition and on the other side, you have your ability to manage it; what makes it harder to keep this balanced? *Prompts: Symptoms, healthcare system navigation, etc… making it harder. Intrinsic strength, social support, spirituality makes it easier*. - What are the key things that improve/decrease/change your ability cope in daily life? (*Probe using responses from questionnaires, and external resources from Q3*) |
|  | Resources: relationship to healthcare professionals | - How would you describe your experience with the doctor/nurse specialist who helps you with your heart condition? Have you ever felt like they don’t listen to the symptoms you report? Tell me about that. (*If present use examples from questionnaire responses*) |
|  | Resources: social support | - Do you get help in managing your heart condition from others (family or friends)? Can you tell me about that? Is there anything that makes this harder/easier? *Prompt: Tension in your relationship, changes to relationship due to decrease in ability.* (*If present use examples from questionnaire responses*) |
|  | Resources: emotional capacity through illness management | - Some people with your heart condition can find the tasks/work they have to do is emotionally stressful, have you felt this way? Can you tell me about that? *Probe as needed* |
|  | Resources: finances in illness management | - Has your heart condition affected you financially? *Prompts: pay for medications, buy healthy food, pay for help, pay for exercise/gym membership, not able to work due to symptoms.* |

| **BoT**  **Domain** | **Theme** | **SYMPACT Phase II Interview schedule** |
| --- | --- | --- |
| **Impact** | Self-value (What can I do? Who am I? Things that make it harder to be me) | - Does your heart condition impact on your life? If yes, how much. If no, why do you think that is? (*use PETS responses as probes*) - Has your heart condition changed your role in life? If yes, how much. If no, why do you think that is? *Prompt: Change in relationships, employability, in recreational activities, in self. Time and energy spent on health (Use PETS and MLHFQ responses)* - Do you always do (miss/skip/forget) what you are supposed to do to manage your heart condition? If yes what and why? *Prompt: forgot, inconvenience of treatment, unwanted side effects.* |
| **Conclusion** | Thoughts on BoT | - Thinking about this idea of the (work and) impact of managing illness, do you think this is true to your experience? |
|  | Final insights | - *Review highlights of what was said:* Have I understood you correctly? Is there anything I haven’t understood? - Is there anything you think I have missed? Anything else you would like me to know about how you go about managing your condition and what makes it harder or easier? - What is the one thing that you wish the doctors/nurses would help you with more? - What is the one thing that you feel would make it easier for you to do the tasks of managing your heart condition? - Thank you for participating and reassure confidentiality |
